# Psychological distress of patients with end-stage kidney disease undergoing dialysis during the 2019 coronavirus disease pandemic: A cross-sectional study in a University Hospital

**DOI:** 10.1371/journal.pone.0260929

**Published:** 2021-12-03

**Authors:** Jin Young Yu, Ji Sun Kim, Chae-Min Hong, Ka Young Lee, Nam-Jun Cho, Samel Park, Hyo-Wook Gil, Eun Young Lee

**Affiliations:** 1 Department of Internal Medicine, Soonchunhyang University Cheonan Hospital, Cheonan, Korea; 2 Department of Psychiatry, Soonchunhyang University Cheonan Hospital, Cheonan, Korea; 3 Department of Medicine, Soonchunhyang University College of Medicine, Cheonan, Korea; 4 Institute of Tissue Regeneration, College of Medicine, Soonchunhyang University, Cheonan, Korea; 5 BK21 Four Project, College of Medicine, Soonchunhyang University, Cheonan, Korea; Ohio State University, UNITED STATES

## Abstract

**Introduction:**

Previous studies have revealed that the COVID-19 pandemic can cause psychological distress such as depression and anxiety. Patients with chronic kidney disease (CKD) might be more vulnerable to psychological distress due to the COVID-19 pandemic. Its impact could be different according to dialysis modality. The aim of this study was to investigate COVID-19-related psychological stress experienced by end-stage kidney disease (ESKD) patients and identify differences in concerns about COVID-19 between hemodialysis (HD) and peritoneal dialysis (PD) patients.

**Methods:**

This cross-sectional study included 148 dialysis patients at Soonchunhyang University Cheonan Hospital from August 2020 to September 2020. These patients responded to a questionnaire covering mental health status and COVID-19 related concerns. Symptoms of depression, anxiety, stress, and insomnia were measured using a 9-item Patient Health Questionnaire (PHQ-9), a 7-item Generalized Anxiety Disorder (GAD-7) scale, a 22-item Impact of Event Scale-Revised (IES-R), and a 7-item Insomnia severity Index (ISI), respectively. Outcomes of HD and PD patients were compared by propensity score matching analysis.

**Results:**

Dialysis patients reported psychological distress including symptoms of depression, anxiety, stress, and insomnia. HD patients showed higher scores for depression (*p* = 0.018), anxiety(*p* = 0.005), stress(*p*<0.001), and insomnia(*p* = 0.006) than the PD patients. After propensity score matching, HD was associated with depression(*p* = 0.0131), anxiety(*p* = 0.0143), and stress(*p* = 0.000415).

**Conclusion:**

Dialysis patients showed psychological distress during the COVID-19 pandemic period, with HD patients having more severe symptoms than PD patients.

## Introduction

In December 2019, a novel coronavirus called severe acute respiratory syndrome coronavirus 2 (SARS-CoV-2) caused an outbreak of coronavirus disease 2019 (COVID-19). In March 2020, World Health Organization (WHO) determined that COVID-19 could be characterized as a pandemic [1]. This crisis has profoundly affected all aspects of the society, including the psychological well-being of people [2]. Previous studies have revealed psychological impact of COVID-19 on anxiety, depressive symptoms, and sleep quality depending on demographic factors and preexisting psychiatric illnesses [3, 4].

Chronic medical comorbidity might play a role in the psychological impact of COVID-19. Patients with chronic medical diseases are more susceptible to contracting COVID-19. They also have higher risk of experiencing severe COVID-19 infection-related complications with poorer prognosis than healthy controls [5]. They also have great fears that a collapse of the healthcare system by COVID-19 may prevent them from receiving appropriate and timely treatment [6].

Likewise, patients undergoing dialysis can have psychological distress during this outbreak. It is known that chronic kidney disease (CKD) is associated with depression and anxiety, making patients more vulnerable to COVID-19-related stress [7–9]. Moreover, patients with CKD have an increased risk of severe COVID-19 infection-related complications and poorer prognosis including higher risks of hospitalization, intensive unit admission, mechanical ventilation, and death [10, 11].

In addition, difference in dialysis modalities can affect the psychological impact of COVID-19 on dialysis patients [12]. For example, patients undergoing hemodialysis are highly dependent on medical institutions. They have to stay in the dialysis center for four hours three times weekly, lying in bed next to each other. Most dialysis centers perform standard precautions for infection control. Droplet-mediated viral infections can spread widely when patients undergo hemodialysis. If one patient in the dialysis center is diagnosed with COVID-19, all members of the dialysis center might have to be isolated. Thus, the Korean Society of Nephrology made a policy allowing only patients with body temperatures of less than 37.5°C without symptoms of COVID-19 to enter the dialysis center [13]. As a result, mild symptoms can delay hemodialysis until the patient is proven to be negative for COVID-19 by real-time polymerase chain reaction (RT-PCR) analysis. In contrast, patients undergoing PD usually visit the hospital once a month unless they have special problems because they are trained to perform PD on their own at home. In terms of receiving dialysis, HD patients are more dependent on medical institutions while PD patients play an important role in dialysis themselves.

In this study, we aimed to investigate COVID-19-related psychological stress experienced by dialysis patients and differences in concerns about COVID-19 between HD and PD patients. End-stage kidney disease (ESKD) patients were defined as those who had kidney function less than 15ml/min/1.73m^2^ classified by CKD grade 5 who needed kidney replacement therapy such as kidney transplantation or dialysis. Our participants were patients with ESKD undergoing dialysis. They decided their dialysis modalities after receiving balanced information [14].

We hypothesized that patients with dialysis would show severe psychological distress and concerns related to COVID-19 and that the dialysis modality would affect the psychological impact of COVID-19.

## Methods study design and participants

This was a cross-sectional, observational study conducted at Soonchunhyang University Hospital Cheonan. The survey was conducted from August 2020 to September 2020 when monthly number of COVID-19 diagnosis cases had reached an all-time high since COVID-19 was found in Korea (3,865 cases in August and 5,642 cases in September). The Institutional Review Board and Ethics Committee of Soonchunhyang University Hospital Cheonan approved this study (IRB number: 2020-07-029). Written informed consent was obtained from each patient before enrollment in this study.

**Fig 1:**
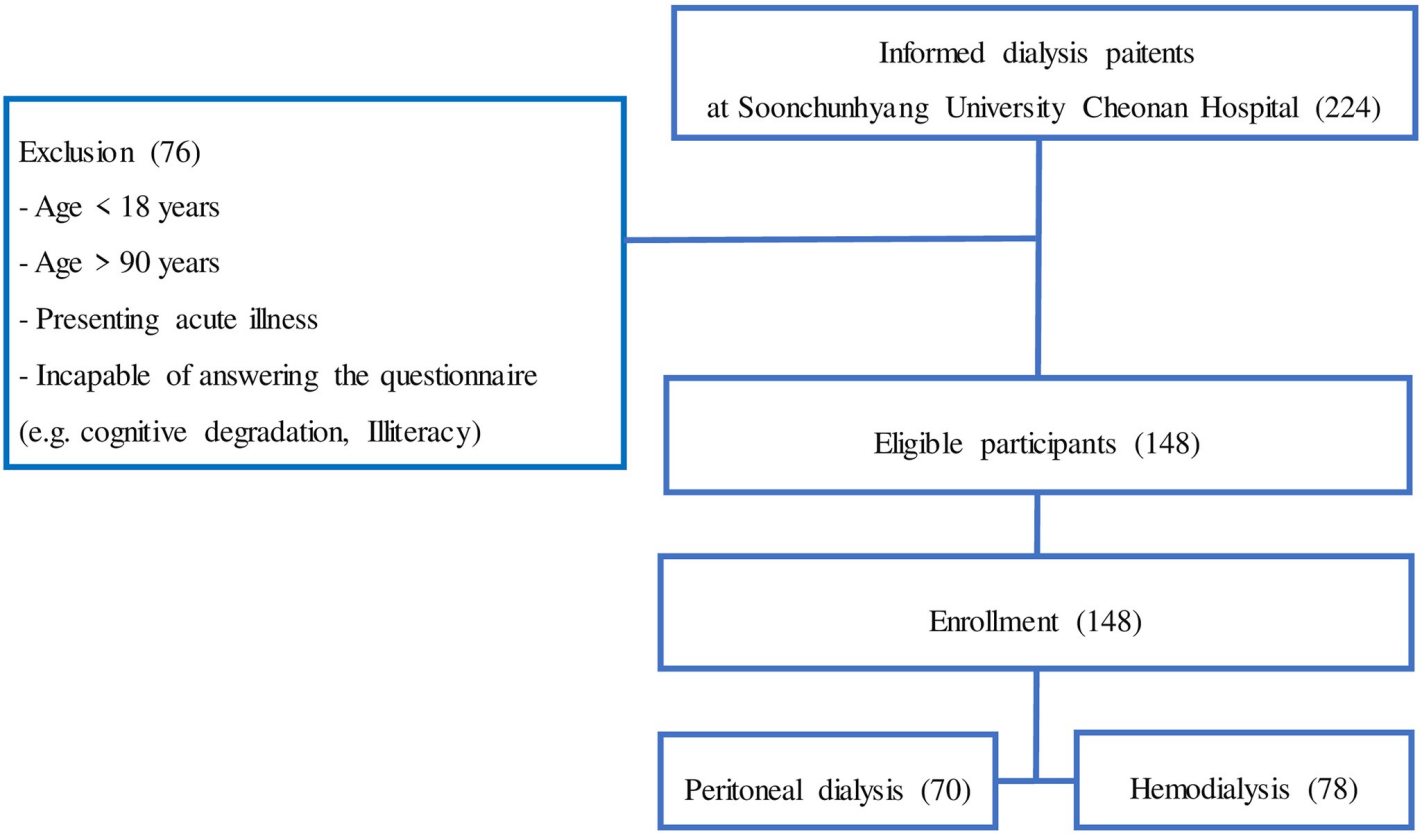
Enrollment flowchart.

There were 224 outpatients undergoing both HD and PD in our artificial kidney center. Artificial kidney center doctors verbally explained this study and gave information about this study to potential participants. Among potential participants, those aged from 18 to 90 years with cognitive ability to respond to the questionnaire were included in this study. Patients with hospitalization within three months or those presenting with acute illness were excluded (Fig 1).

### Survey development

We reviewed medical records and analyzed laboratory data to assess whether dialysis was performed adequately. Information on the causative disease of dialysis and dialysis vintage were obtained by reviewing medical records. Dialysis adequacy was evaluated by the single pool Kt/V. Information about the severity of anemia, electrolyte imbalance, and CKD-mineral bone disease (CKD-MBD) were also collected.

A structured questionnaire consisted of questions covering mental health status, concerns about COVID-19, precautionary measures taken against COVID-19, and the psychological impact of COVID-19.

Mental health status was assessed by the degree of symptoms related to depression, anxiety, insomnia, and distress. We adopted the 9-item Patient Health Questionnaire (PHQ-9) [15], the 7-item Generalized Anxiety Disorder(GAD-7) scale [16], the 7-item Insomnia severity Index [17], and the 22-item Impact of Event Scale-Revised(IES-R) [18] to measure the severity of depression, anxiety, insomnia, and distress, respectively Questionnaires measuring scales were provided by a hard copy. Total scores of measurement tools were interpreted as follows: PHQ-9, normal (0–4), mild (5–9), moderate (10–14), and severe (15–21) depression; GAD-7, normal (0–4), mild (5–9), moderate (10–14), and severe (15–21) anxiety; ISI, normal(0–7), mild (8–14), moderate (15–21), and severe (22–28) insomnia; and IES-R, normal (0–8), mild (9–25), moderate (26–43), and severe (44–88) distress. These categories were based on values established in the literature which was undertaken in a sample of the general population in China [19].

We analyzed the correlations between concerns and precautionary measures taken for COVID-19 and the psychological impact of the outbreak. Questions about concerns and precautionary measures related to COVID-19, validated in a previous study in a sample of the general population in China [19] were adopted. Questionnaires about concerns and precautionary measures for COVID-19 were provided by papers.

Concerns about COVID-19 included four questions: (1) the likelihood of contracting COVID-19 during the current outbreak, (2) the level of confidence in own doctor’s ability to diagnose or recognize COVID-19, (3) the likelihood of surviving if infected with COVID-19, and (4) concerns about other family members getting COVID-19 infections [19].

Precautionary measures taken against COVID-19 included: (1) covering mouth when coughing and sneezing; (2) avoiding sharing utensils(e.g., chopsticks) during meals; (3) washing hands immediately after coughing, rubbing the nose, or sneezing; (4) wearing a mask regardless of the presence or absence of symptoms; (5) the average number of hours staying at home per day to avoid COVID-19; and (6) feeling that too much unnecessary worry has been made about the COVID-19 outbreak [19].

### Statistical analysis

Independent sample t-test was used to compare demographic and psychological characteristics between HD and PD groups. Chi-squared test or Fisher’s exact test was used to compare categorical data. Correlation analyses were performed to examine the relationship between concerns and precautionary measures taken for COVID-19 and the psychological impact of the outbreak. We additionally conducted linear regression analysis for COVID-19-associated psychological distress (depression, anxiety, and stress) and concerns and precautionary measures taken for COVID-19. All statistical analyses were performed using SPSS 21 (SPSS, Inc., Chicago, IL, USA). The significance level was set at *p*<0.05.

We performed propensity score matching analysis because of unbalanced baseline characteristics between HD and PD groups [20, 21]. Propensity scores were calculated using multivariable logistic regression to estimate the probability of receiving PD versus HD. Nearest-neighbor matching without replacement was used with a caliper distance of 0.2 after the order of HD and PD groups was randomized [21]. Standardized differences were used to evaluate the balance in the distribution of baseline variables between HD and PD groups in the matched cohort, where a difference of <0.2 was taken to indicate a sufficient balance [20]. Multiple linear regression stratified by matched pairs was conducted to examine the association between dialysis type and test scores [21].

## Results

### Participants

Of 224 patients undergoing dialysis at Soonchunhyang University Cheonan Hospital, 148 patients were eligible. All eligible participants volunteered to participate in this study. Among these participants, 78 patients were undergoing HD and 70 patients were undergoing PD (Fig 1). During the survey, they all maintained their dialysis treatment as usual. In the HD group, 67.9% were males. In the PD group, 50% were males. The mean age of patients was 55.32 years in the HD group and 51.44 years in the PD group (*p* = 0.06). The median dialysis vintage for HD patients was around 6.77 years, which was longer than that (4.37 years) for PD patients (*p* = 0.0001). We implemented square root transformation for variables that having standard normal variations. Both groups of patients had been receiving dialysis adequately when assessed by indicators including Kt/V, anemia profile, and electrolytes. Causes of kidney disease showed similar distributions in each group.

To overcome unbalanced baseline characteristics such as age, sex, and dialysis vintage, propensity score-matching was performed, leading to 52 pairs of matched patients. After propensity score matching, both groups of patients were well-balanced as shown in Table 1.

**Table 1:** Baseline characteristics of patients in this study.

	Whole cohort			Matched cohort		
	HD (*n* = 78)	PD (*n* = 70)	*P*	HD (*n* = 52)	PD (*n* = 52)	*p*
**Age (years)**	55.3 ± 12.4	51.4 ± 12.0	0.056	56.1 ± 12.6	54.1 ± 10.6	0.401
**Sex**			0.040			1.000
**Male**	53 (67.9)	35 (50.0)		29	29	
**Female**	25 (32.1)	35 (50.0)		23	23	
**BMI (kg/m** ^**2**^ **)**	24.2±3.8	24.8±4.2	0.360	24.3 ±4.2	24.4 ±4.0	0.889
**Kidney disease**			0.543			0.696
**Diabetic nephropathy**	25	24		22	18	
**Hypertensive disease**	28	26		17	20	
**Glomerulonephritis/vasculitis**	19	19		10	13	
**Cystic/hereditary/congenital disease**	3	0		1	0	
**Etiology unknown or missing**	3	1		2	1	
**Dialysis duration (years)**	6.77 (2.81, 13.36)	4.37 (1.47, 6.47)	0.0001	4.13 (2.35, 7.88)	4.95 (2.23, 7.18)	0.709
**Kt/V**	1.73 (1.50, 2.07)	1.95 (1.71, 2.36)	0.004	1.72 (1.48, 2.15)	1.95 (1.71, 2.28)	0.045
**Biochemical parameters (serum)**						
**Hemoglobin (g/dL)**	10.8±1.5	10.2±1.6	0.030	10.5 ±1.2	10.6 ±1.5	0.602
**Hematocrit (%)**	32.0±4.8	30.3±4.7	0.033	31.2 ±3.8	31.4 ±4.7	0.732
**Sqrt_ferritin**	14.8±5.0	20.6±7.2	< 0.001	15.3±4.7	20.8±7.6	<0.001
**Sqrt_TSAT**	5.53+1.17	6.17+1.29	0.002	5.48 ±1.18	6.27 ±1.25	0.001
**Creatinine (mg/dL)**	10.4±3.2	10.5±4.1	0.856	9.4 ±3.1	10.1 ±3.9	0.356
**Phosphorous (mg/dL)**	4.92±1.75	5.14±1.38	0.413	4.98 ±1.76	4.91 ±1.32	0.885
**Calcium (mg/dL)**	8.91±0.64	9.17±1.01	0.067	8.90 ±0.53	9.34 ±1.02	0.008
**Potassium (mmol/L)**	4.71±0.65	4.30±0.76	< 0.001	4.72 ±0.63	4.28 ±0.83	0.003
**Sqrt_intact PTH (pg/mL)**	17.3+5.0	16.5+5.8	0.344	17.4 ±5.3	15.9 ±5.7	0.149

HD: hemodialysis; PD: peritoneal dialysis; Sqrt: square root transformation; BMI: body mass index; TSAT: transferrin saturation; PTH: parathyroid hormone.

### Psychological measurements

HD patients showed significant psychological distress. All scores reflecting depression (PHQ-9), anxiety (GAD-7), stress (IES-R), and insomnia (ISI) were higher in HD patients than in PD patients (Table 2). About 30.7% of HD patients and 15.8% of PD patients reported moderate to severe depression, showing significant (*p* = 0.001) difference between the two. Nearly half of HD patients and 28.5% of PD patients reported moderate to severe anxiety, showing no significant (*p* = 0.081) difference between the two. More than half of PD patients and 25.6% of HD patients had a normal range of IES-R (*p*<0.001). About 65.7% of PD patients and 38.5% of HD patients had a normal range of ISI (*p* = 0.002) (Table 2).

**Table 2:** Comparison of psychological characteristics between HD and PD patients.

	HD patients (N = 78)	PD patients (N = 70)	*P*
**PHQ-9**	6.76 ± 6.45	4.29 ± 6.06	0.018
Severity			0.001
normal	35 (44.9)	51 (72.9)	
mild	19 (24.4)	8 (11.4)	
moderate	14 (17.9)	2 (2.9)	
severe	10 (12.8)	9 (12.9)	
**GAD-7**	11.59 ± 5.91	9.31 ± 3.95	0.005
Severity			0.081
normal	0 (0)	1 (1.4)	
mild	41 (52.6)	49 (70.0)	
moderate	19 (24.4)	12 (17.1)	
severe	18 (23.1)	8 (11.4)	
**IES-R**	19.10 ± 14.84	10.57 ± 11.55	< 0.001
Severity			< 0.001
normal	20 (25.6)	42 (60.0)	
mild	33 (42.3)	17 (24.3)	
moderate	19 (24.4)	9 (12.9)	
severe	6 (7.7)	2 (2.9)	
**ISI**	8.88 ± 5.50	6.76 ± 5.83	0.006
Severity			0.002
normal	30 (38.5)	46 (65.7)	
mild	36 (46.2)	16 (22.9)	
moderate	12 (15.4)	6 (8.6)	
Severe	0 (0)	2 (2.9)	

HD: hemodialysis; PD: peritoneal dialysis; PHQ-9: the 9-item Patient Health Questionnaire; GAD-7: the 7-item Generalized Anxiety Disorder scale; IES-R: the 22-item impact of Event Scale-Revised; ISI: insomnia severity index.

After propensity score matching, PHQ-9, GAD-7, and IES-R scores were associated with the dialysis modality. HD patients had higher PHQ-9, GAD-7, and IES-R scores in the matched cohort (Table 3).

**Table 3:** Psychological characteristics after PSM analysis.

	Estimated	Standard error	T value	Pr (>|t|)
3–1. Depression (PHQ-9)				
Intercept	3.11730	6.85937	0.454	0.6505
Group (HD)	3.36699	1.33283	2.526	0.0131*
Age	0.03731	0.05596	0.667	0.5066
Sex (female)	1.19719	1.35198	0.886	0.3781
Hb	-0.3631	0.47516	-0.764	0.4465
Dialysis vintage	-0.08975	0.13629	-0.658	0.5118
Sqrt_ferritin	0.1482	0.10117	1.465	0.1461
3–2. Anxiety (GAD7)				
Intercept	12.29244	5.61950	2.187	0.0311*
Group (HD)	2.72374	1.09191	2.494	0.0143*
Age	0.04469	0.04585	0.975	0.3321
Sex (female)	1.89810	1.10761	1.714	0.0898
Hb	-0.50174	0.38928	-1.289	0.2005
Dialysis vintage	-0.23408	0.11166	-2.096	0.0386*
Sqrt_ferritin	0.01621	0.08288	0.196	0.8454
3–3. Stress (IES-R)				
Intercept	5.3586	14.9860	0.358	0.721439
Group (HD)	10.6478	2.9119	3.657	0.000415***
Age	0.1221	0.1223	0.999	0.320356
Sex (female)	1.0765	2.9538	0.364	0.716311
Hb	-0.4678	1.0381	-0.451	0.653232
Dialysis vintage	-0.3557	0.2978	-1.194	0.235195
Sqrt_ferritin	0.2319	0.2210	1.049	0.296709
3–4. Insomnia (ISI)				
Intercept	6.13766	6.67457	0.920	0.3601
Group (HD)	2.41568	1.29692	1.863	0.0655
Age	-0.02545	0.05446	-0.467	0.6413
Sex (female)	2.21071	1.31556	1.680	0.0961
Hb	0.02079	0.46236	0.045	0.9642
Dialysis vintage	-0.01543	0.13262	-0.116	0.9076

PSM: propensity score matching; HD: hemodialysis; PD: peritoneal dialysis; PHQ-9: the 9-item Patient Health Questionnaire; GAD-7: the 7-item Generalized Anxiety Disorder scale; IES-R: the 22-item impact of Event Scale-Revised; ISI: insomnia severity index; Sqrt: square root transformation; Hb: hemoglobin.

### Concerns about COVID-19

Concerns about COVID-19 for HD and PD patients are shown in Table 4. Table 5 shows the correlation between psychological distress and concerns in the whole cohort. Results of analysis for each dialysis group are shown in Table 6.

**Table 4:** Comparison of concerns and precautionary measures for 2019 coronavirus disease (COVID-19) between HD and PD patients.

	N (%) or mean (SD)	Whole cohort			Matched cohort		
		HD patients (*n* = 78)	PD patients (*n* = 70)	*p*	HD patients (*n* = 52)	PD patients (*n* = 52)	*p*
Concerns	Likelihood of contracting COVID-19 during the current outbreak						
	Very likely	1 (1.3)	0 (0)	0.007	1 (1.9)	0 (0)	< 0.001
	Somewhat likely	2 (2.6)	2 (2.9)		1 (1.9)	1 (1.9)	
	Not very likely	43 (55.1)	20 (28.6)		34 (65.4)	16 (30.8)	
	Not likely at all	32 (41)	48 (68.6)		16 (30.8)	35 (67.3)	
	Level of confidence in patient’s own doctor’s ability to diagnose or recognize						
	Very confident	16 (20.5)	34 (48.6)	0.001	9 (17.3)	29 (55.8)	< 0.001
	Somewhat confident	59 (75.6)	32 (45.7)		41 (78.8)	19 (36.5)	
	Not very confident	0 (0)	2 (2.9)		0 (0.0)	2 (3.8)	
	Not at all confident	3 (3.8)	2 (2.9)		2 (3.8)	2 (3.8)	
	Likelihood of surviving if infected with COVID-19						
	Very likely	7 (9.0)	15 (21.4)	0.039	4 (7.7)	14 (26.9)	0.019
	Somewhat likely	28 (35.9)	30 (42.9)		19 (36.5)	19 (36.5)	
	Not very likely	37 (47.4)	19 (27.1)		25 (48.1)	13 (25.0)	
	Not likely at all	6 (7.7)	6 (8.6)		4 (7.7)	6 (11.5)	
	Concerns about other family members getting COVID-19 infections						
	Very likely	29 (37.2)	20 (28.6)	0.339	21 (40.4)	12 (23.1)	0.128
	Somewhat likely	39 (50.0)	41 (58.6)		24 (46.2)	31 (59.6)	
	Not very likely	6 (7.7)	8 (11.4)		4 (7.7)	8 (15.4)	
	Not likely at all	4 (5.1)	1 (1.4)		3 (5.8)	1 (1.9)	
Precautionary measures	Covering mouth when coughing and sneezing						
	Very likely	23 (29.5)	32 (45.7)	0.238	16 (30.8)	22 (42.3)	0.757
	Somewhat likely	48 (61.5)	33 (47.1)		32 (61.5)	27 (51.9)	
	Not very likely	6 (7.7)	4 (5.7)		3 (5.8)	2 (3.8)	
	Not likely at all	1 (1.3)	1 (1.4)		1 (1.9)	1 (1.9)	
	Avoiding sharing utensils (e.g., chopsticks) during meals						
	Very likely	13 (16.7)	16 (22.9)	0.608	10 (19.2)	12 (23.1)	0.871
	Somewhat likely	42 (53.8)	35 (50.0)		29 (55.8)	26 (50.0)	
	Not very likely	18 (23.1)	17 (24.3)		12 (23.1)	12 (23.1)	
	Not likely at all	5 (6.4)	2 (2.9)		1 (1.9)	2 (3.8)	
	Washing hands immediately after coughing, rubbing the nose, or sneezing						
	Very likely	9 (11.5)	21 (30.0)	0.012	7 (13.5)	14 (26.9)	0.166
	Somewhat likely	38 (48.7)	33 (47.1)		25 (48.1)	26 (50.0)	
	Not very likely	30 (38.5)	14 (20.0)		19 (36.5)	11 (21.2)	
	Not likely at all	1 (1.3)	2 (2.9)		1 (1.9)	1 (1.9)	
	Wearing mask regardless of the presence or absence of symptoms						
	Very likely	33 (42.3)	48 (68.6)	0.004	21 (40.4)	34 (65.4)	0.018
	Somewhat likely	39 (50.0)	22 (31.4)		29 (55.8)	18 (34.6)	
	Not very likely	5 (6.4)	0 (0)		1 (1.9)	0 (0)	
	Not likely at all	0 (0)	0 (0)		0 (0)	0 (0)	
	Feeling that too much unnecessary worry surrounds the COVID-19 outbreak						
	Very likely	9 (11.5)	13 (18.6)	0.060	6 (11.5)	11 (21.2)	0.164
	Somewhat likely	35 (44.9)	27 (38.6)		23 (44.2)	18 (34.6)	
	Not very likely	28 (35.9)	30 (42.9)		20 (38.5)	23 (44.2)	
	Not likely at all	6 (7.7)	0 (0.0)		3 (5.8)	0 (0.0)	
	Average number of hours stayed at home per day to avoid COVID-19						
	Hours	14.73 ± 6.28	17.23 ± 5.95	0.033	15.4±5.7	17.4±6.0	0.081

**Table 5:** Correlation between concerns and precautionary measures for the 2019 coronavirus disease (COVID-19) and the psychological impact of the outbreak on dialysis patients.

	*r*			
	Depression	Anxiety	Stress	Insomnia
Dialysis modality (HD)	0.194*	0.22**	0.305**	0.186*
Age	0.18*	0.197*	0.183*	0.034
Dialysis vintage	0.051	-0.056	0.004	0.135
Concerns				
1. Likelihood of contracting COVID-19 during the current outbreak	-0.079	-0.218**	-0.256**	-0.133
2. Level of confidence in patient’s own doctor’s ability to diagnose or recognize	0.232**	0.199*	0.228**	0.003
3. Likelihood of surviving if infected with COVID-19	0.279**	0.186*	0.235**	0.199*
4. Concerns about other family members getting COVID-19 infections	-0.205*	-0.24**	-0.248**	-0.149
Precautionary measures				
5. Covering mouth when coughing and sneezing	0.003	-0.01	0.059	0.031
6. Avoiding sharing utensils (e.g., chopsticks) during meals	0.043	0.033	0.068	0.027
7. Washing hands immediately after coughing, rubbing the nose, or sneezing	0.12	0.054	0.125*	0.049
8. Wearing mask regardless of the presence or absence of symptoms	0.085	0.088	0.176*	0.163*
9. Feeling that too much unnecessary worry surrounds the COVID-19 outbreak	-0.193*	-0.245**	-0.279**	-0.28**

*p< 0.05,

**p< 0.01.

**Table 6:** Correlation between concerns and precautionary measures for the 2019 coronavirus disease (COVID-19) and the psychological impact of the outbreak on dialysis patients.

		Concerns and precautionary measures for COVID-19	*r*			
			Depression	Anxiety	Stress	Insomnia
HD patients (N = 78)	Concerns	1. Likelihood of contracting COVID-19 during the current outbreak	0.086	-0.108	-0.102	-0.003
		2. Level of confidence in patient’s own doctor’s ability to diagnose or recognize	0.320**	0.257*	0.254*	0.031
		3. Likelihood of surviving if infected with COVID-19	0.142	0.092	0.062	0.079
		4. Concerns about other family members getting COVID-19 infections	-0.104	-0.176	-0.168	-0.116
	Precautionary Measures	5. Covering mouth when coughing and sneezing	0.004	0.045	0.155	0.114
		6. Avoiding sharing utensils (e.g., chopsticks) during meals	-0.116	-0.105	-0.081	-0.072
		7. Washing hands immediately after coughing, rubbing the nose, or sneezing	-0.016	-0.047	0.034	-0.032
		8. Wearing mask regardless of the presence or absence of symptoms	-0.041	-0.025	0.111	0.126
		9. Feeling that too much unnecessary worry surrounds the COVID-19 outbreak	-0.305**	-0.368**	-0.386**	-0.446**
PD patients (N = 70)	Concerns	1. Likelihood of contracting COVID-19 during the current outbreak	-0.188	-0.298*	-0.343**	-0.194
		2. Level of confidence in patient’s own doctor’s ability to diagnose or recognize	0.080	0.036	0.089	-0.100
		3. Likelihood of surviving if infected with COVID-19	0.368**	0.252*	0.359**	0.254*
		4. Concerns about other family members getting COVID-19 infections	-0.342**	-0.368**	-0.389**	-0.185
	Precautionary Measures	5. Covering mouth when coughing and sneezing	-0.056	-0.182	-0.167	-0.102
		6. Avoiding sharing utensils (e.g., chopsticks) during meals	0.204	0.227	0.236*	0.104
		7. Washing hands immediately after coughing, rubbing the nose, or sneezing	0.179	0.079	0.097	0.045
		8. Wearing mask regardless of the presence or absence of symptoms	0.137	0.134	0.068	0.103
		9. Feeling that too much unnecessary worry surrounds the COVID-19 outbreak	-0.108	-0.119	-0.240*	-0.149

*p< 0.05,

**p< 0.01.

Most participants thought that they were not likely to be exposed to COVID-19 during the current outbreak. More HD patients answered “not likely at all” than PD patients in the matched cohort, showing a significant (*p*<0.001) difference between the two. Symptoms of anxiety and stress were associated with concerns about possible viral exposure in the whole cohort. Similar results were found when PD patients were analyzed separately (Table 6). More PD patients answered that they would “very likely” or “somewhat likely” survive during a COVID-19 outbreak than HD patients in the matched cohort, showing a less significant (*p* = 0.019) difference. Symptoms of depression, anxiety, stress, and insomnia were correlated with concerns about survival in all participants (Table 5). Similar results were found when PD patients were analyzed separately (Table 6). More PD patients answered they were "very confident" in their own doctor’s ability to recognize and diagnose COVID-19 than HD patients in the matched cohort, showing a significant (*p*<0.001) difference between the two. The level of confidence in their doctors was related to the patient’s psychological distress including depression, anxiety, and stress in all patients (Table 5). Similar results were found in HD patients (Table 6). There was no significant difference in concerns about other family members getting COVID-19 infections between HD and PD patients. Most participants answered “very likely” or “somewhat likely” about worries about their families. It was associated with symptoms of depression, anxiety, and stress in all participants (Table 5). More than half in both HD and PD groups of patients felt too much unnecessary worries about COVID-19. Symptoms of depression, anxiety, stress, and insomnia were correlated with this feeling of unnecessary worry in all participants (Table 5).

### Precautionary measures

We compared precautionary measures taken for COVID-19 between HD and PD patients. Results are shown in Table 4. Table 5 shows results of correlation between psychological distress and precautionary measures in the whole cohort. We also analyzed each group separately. Results are shown in Table 6. There was no significant difference in precautionary measures except for the compliance of wearing masks between HD and PD patients. PD patients had better compliance of wearing masks regardless of symptoms than HD patients in the matched cohort, showing a less significant (*p* = 0.018) difference between the two. PD patients answered either “very likely” (68.6%) or “somewhat likely”(31.4%) to the question about wearing masks. In contrast, 6.4% (*n* = 5) of HD patients answered “not very likely.” Symptoms of stress and insomnia were associated with wearing masks in the whole cohort (Table 5). More PD patients tended to self report adherence to cough etiquette and hand hygiene than HD patients, although the difference between the two was not statistically significant. Of PD patients, 45.7% answered "very likely" to the question about whether they covered their mouths when coughing and sneezing, whereas only 29.5% of HD patients did so (*p* = 0.757). Regarding hand hygiene, 26.9% of PD patients and 13.5% of HD patients answered “very likely” about washing hands (*p* = 0.166). There was no significant difference patients in the habit of sharing utensils (e.g., chopsticks) during meals between HD and PD patients. The average time staying at home to avoid COVID-19 was not significantly different between HD and PD patients, either. In HD patients, linear regression analysis with depression as a dependent variable showed significant associations with sex, the “level of confidence in the doctor’s ability,” and “feelings that too much unnecessary worry has been made about the COVID-19 outbreak” (R^2^ = 0.247, F = 7.875, *p* <0.001, standardized β = 0.229, t = 2.212, *p* = 0.030; standardized β = 0.297, t = 2.893, *p* = 0.005; and standardized β = -0.280, t = -2.711, *p* = 0.008, respectively). The model for anxiety also showed significant associations with sex and the above two concerns (R^2^ = 0.277, F = 9.216, *p*<0.001, standardized β = 0.283, t = 2.788, *p* = 0.007; standardized β = 0.227, t = 2.251, *p* = 0.027; and standardized β = -0.332, t = -3.286, *p* = 0.002, respectively). In PD patients, the linear regression analysis with depression as a dependent variable showed significant associations with the “likelihood of surviving if infected with COVID-19” and “concerns about other family members getting COVID-19 infections” (R^2^ = 0.208, F = 8.793, *p*<0.001, standardized β = 0.309, t = 2.774, *p* = 0.007; and standardized β = -0.276, t = -2.479, *p* = 0.016, respectively).

## Discussion

In this study, we investigated COVID-19-related psychological stress experienced by dialysis patients and the difference in concerns between HD and PD patients. First, dialysis patients had relatively high psychological distress during the COVID-19 pandemic period. Second, HD patients showed higher psychological distress and concerns related to COVID-19 than PD patients. Third, various concerns and precautionary measures for COVID-19 were associated with the psychological distress of HD and PD patients. In a previous study that meta-analyzed factors associated with psychological distress, pre-existing physical conditions with higher COVID-19 infection risk were associated with higher anxiety and depression [22], consistent with results of the present study showing a high degree of anxiety and depression symptoms and psychological distress in dialysis patients during the COVID-19 pandemic period. Sources of distress might include feelings of vulnerability or the loss of control and concerns about one’s health and the spread of the virus [23]. The fact that COVID-19 is transmitted human-to-human and that it is associated with high morbidity might intensify the perception of personal health problems, especially for patients with chronic illnesses [24, 25]. However, due to the sudden occurrence of this outbreak, we were unable to assess an individual’s psychological condition before the outbreak. Since there were no data before COVID-19, it was difficult to conclude that the more pronounced psychological distress of HD patients was only due to COVID-19. Interestingly, a recent study in the Netherlands [26] investigating the mental health of dialysis patients (on HD, PD, and home hemodialysis [HHD]) during COVID-19 and compared with the period preceding the pandemic has reported the mental health of dialysis patients is unaffected by the pandemic. To overcome this limitation, we included concerns and precautionary measures taken for COVID-19 and showed their associations with the psychological distress of patients. We also tried to identify the difference between HD and PD patients. They decided their dialysis modalities with balanced information. As a result, they have a unique medical behavior. Thus, it is worth to investigate how dialysis modalities work in the COVID-19 era. According to our study, the psychological distress of COVID-19 was more pronounced in HD patients than in PD patients. Interestingly, in our regression analysis, HD patients had lower level of confidence. This can be related to the severity of their depressive and anxiety symptoms. The nature of patients undergoing HD being highly dependent on the hospitals and doctors might have an impact on the above results. Regarding concerns about COVID-19, more than half of both HD and PD patients felt there was too much unnecessary worry about COVID-19. Chronic disease patients experienced psychological distress more than the general population in previous studies [22, 27], similar to our results. In addition, compared to PD patients, more HD patients responded that they felt less likely to be exposed to the virus. However, only a few of them answered that they would survive if infected with COVID-19. These results suggest that HD patients might have cognitive bias related to risk perception and more emotional impact than PD patients. Cognitive biases are associated with psychopathology. They may alter threat perception, while risk perception is strongly associated with the emotional impact of the threat itself [28]. In terms of precautionary measures, PD patients maintained cough etiquette better with better hand-washing and mask-wearing compliance than HD patients. It maybe because PD patients are educated to always wear masks and perform hand hygiene when exchanging peritoneal fluid. Thus, they have formed such habits. Interestingly, other precautionary measures such as sharing utensils or the average time staying at home did not differ between the two groups. The fact that HD patients need to travel to the dialysis center and stay four hours three times a week and spend the time for hemodialysis itself might have caused the lack of significant difference in the time spent at home. In other words, PD patients showed better compliance only for elements on which they were educated. The self-efficacy of knowing the precautionary measures and being able to do them well might have alleviated the psychological distress for PD patients more than for HD patients. This interpretation can extend to the importance of educating patients on infection control. We expect that if medical staff pay more attention to educating HD patients on precautionary measures for COVID-19, it may help reduce their psychological distress. In PD patients, the likelihood of surviving if infected with COVID-19 and concerns about their family members can affect the severity of their psychological distress. Considering the independent management of dialysis and the confidence of controlling infections related to COVID-19 by the habit of watching for infections in PD patients (PD patients might maintain a higher level of alertness of infection because they always make an effort not to develop peritonitis infections), our results suggest that the depression and anxiety of PD patients might have caused more realistic and practical concerns such as viral exposure and survival from COVID-19 infections. This study had some limitations. First, it was conducted as a cross-sectional, single-center study, making it difficult to make causal inferences. When interpreting our results, the relatively small sample size of this study should be considered. Our results might not be generalized to populations of other ethnicities. Further longitudinal multi-center studies with larger samples are needed. Second, the scales used were self-reported measures and the constructed questions might not reflect all COVID-19-related concerns. We tried to overcome these limitations by using reliable and validated instruments that have been widely used in other studies [12]. COVID-19-related concerns were constructed with reference to prior research in China [12]. Although some modifications were made to suit the current situation in Korea, they might not have fully reflected differences between Korea and China. We understand that it is unusual all eligible patients volunteered to participate in this study. However, the whole process, including recruiting participants, was conducted without coercion. We assumed that the high participation rate was influenced by the fact that it was a non-invasive study and that our participants were outpatients who were able to perform daily activities. It could also be associated with the fact that patients who may feel difficult to participate in the study were excluded by the criteria of having ‘acute illness’ or ‘degraded cognition’. Despite the above limitations, this was the first study to investigate differences in psychological distress and concerns for COVID-19 between HD and PD patients. It also identified risk factors related to psychological distress. As we hypothesized, patients with dialysis had a high degree of psychological distress during the COVID-19 pandemic period. In addition, the dialysis modality made a difference. Our results might offer insight into the necessities of beforehand interventions to prevent psychological distress and depression in dialysis patients who are vulnerable to pandemic infections such as COVID-19.

## Conclusion

Dialysis patients had psychological distress during the COVID-19 pandemic period, with HD patients having more severe symptoms than PD patients. Psychological distress scale scores including PHQ-9, GAD-7, IES-R, and ISI were higher in HD patients than in PD patients after analyzing the whole cohort. After propensity score matching, HD patients showed higher degrees of PHQ-9, GAD-7, and IES-R scores than PD patients. COVID-19-related concerns and precautionary measures were associated with patients’ psychological distress.
